# Awareness of Chronic Obstructive Pulmonary Disease and Its Risk Factors Among the Adult Population of the Qassim Region, Saudi Arabia

**DOI:** 10.7759/cureus.44743

**Published:** 2023-09-05

**Authors:** Ahmed S Almuzaini, Mutab Algeffari, Asma Alsohaibani, Latifah Y Almutlaq, Raghad Alwehaibi, Reema A Almuzaini, Syed E Mahmood

**Affiliations:** 1 Department of Family and Community Medicine, Qassim University, Buraydah, SAU; 2 Department of Family and Community Medicine, King Khalid University, Abha, SAU

**Keywords:** shisha smoker, qassim region, lungs, electronic smoker, chronic obstructive pulmonary disease, cigarette smoker

## Abstract

Background: Epidemiological studies are crucial in appraising the occurrence of chronic obstructive pulmonary disease (COPD) in a specific region, establishing benchmarks, and devising effective preventive measures. It is against this background that the study aims to evaluate adult awareness of COPD and its risk factors among adults in the Qassim Region, Saudi Arabia.

Method: This observational cross-sectional study was conducted in the Qassim Region and involved consenting adults who voluntarily participated. Between 20 May and 4 June 2023, a self-administered online survey was distributed through social media platforms, utilizing an anonymous, self-explanatory questionnaire to evaluate participants' awareness of COPD.

Results: In our study, a total of 1,306 participants were enrolled, of which 27.6% (n=360) reported having ever heard of COPD. Among all respondents, 21.3% (n=278) stated that they study or work in medical-related fields, and out of them, 60.4% (n=168) had prior awareness of COPD. Upon excluding participants associated with medical-related fields, the overall awareness level decreased to 18.7%. The majority of respondents fell within the age range of 18 to 29 years, of whom 34.5% had ever heard of COPD. Regarding smoking habits, the majority were cigarette smokers (38.4%), and of all cigarette smokers, 22.4% had heard of COPD. The second highest proportion of smokers (35.8%) were electronic smokers, and among them, 24.1% were aware of COPD. The lowest proportion of smokers (25.8%) were shisha smokers, with 25.6% of them having heard of COPD. Among the 1,306 respondents, only 27.5% (n=360) had ever heard of COPD. When asked about the organ affected by COPD, 81% (n=292) of those who were aware of the condition correctly responded that it affects the lungs. On the other hand, 8.9% (n=32) incorrectly selected "heart" as the affected organ, while 2.5% (n=9) chose "I don't know," and none selected "throat."

Conclusion: The Qassim Region in Saudi Arabia exhibits a reduced level of COPD awareness among the general population. It is imperative to urgently address this situation and enhance awareness for improved COPD diagnosis and treatment. Considering the region's high prevalence of COPD and associated risk factors, it becomes vital to strengthen educational curricula and integrate COPD awareness into public forums and awareness campaigns. Moreover, conducting additional national research would be instrumental in assisting policymakers in developing effective preventive and therapeutic strategies.

## Introduction

Chronic obstructive pulmonary disease (COPD) is characterized by progressive airflow limitation and tissue destruction. It is associated with irreversible structural lung changes due to chronic inflammation from prolonged exposure to noxious particles or gases most commonly cigarette smoke. However, other environmental factors, such as exposure to indoor air pollutants, might influence COPD risk, particularly in developing countries [1,2].

Patients usually present with chronic and progressive dyspnea, cough, and sputum production. Patients may also have wheezing, chest tightness, and frequent acute exacerbations which are mainly triggered by viral or bacterial infections. In severe cases, this can lead to respiratory failure and pulmonary heart disease [1]. The airflow limitation and tissue destruction were confirmed by spirometry. It includes chronic obstructive bronchiolitis and emphysema, which lead to air trapping and shortness of breath in response to physical exertion [2]. Based on reported symptoms of COPD or diagnosed COPD and smoking history, one population-based study found a COPD prevalence of 3.5% in Middle Eastern populations (2.4% in Saudi populations). The study used spirometry to confirm COPD diagnosis with a sample of Saudi smokers aged >40 in a primary care clinic, and 14.2% of them have COPD [3].

In 2019, COPD emerged as a prevalent health issue, affecting 10.3% of individuals aged 30-79 years globally (95% CI 8.2-12.8) [4]. It is crucial to highlight that COPD has become a significant contributor to morbidity and disability in recent years [5]. Moreover, it is noteworthy that in 2019, COPD ranked as the third leading cause of mortality, resulting in 3.23 million deaths. Furthermore, COPD also holds the seventh position when considering its impact on global health [6]. Most people who die from COPD in low- and middle-income countries are under 70 years old primarily due to the high cost of treatment and limited hospital services, which may raise their risk of disease progression, death, and complications [6].

Despite the limited data that are available, it is believed that the increased prevalence of tobacco smoking among men and women in Saudi Arabia contributes to an increase in COPD prevalence. As smoking accounts for most cases of COPD, over 7,000 chemicals are produced when a cigarette burns, many of which are harmful as they affect the immune system and irritate the lining of bronchioles and alveoli [4].

Saudi Arabian smokers report a low awareness of COPD [7]. This likely contributes to the significant under-diagnosis of the disease, which is evident virtually everywhere in the world, albeit to varying degrees. Health education about COPD is lacking, according to studies, not just among the general population but also among COPD sufferers [8] and even their families [9,10].

Epidemiological studies are crucially needed to assess the prevalence of COPD in the region to determine the baseline, against which the future trends in the risk factor levels can be assessed and preventive strategies be planned to promote health among the population. This study aims to assess the awareness of COPD and its risk factors in the Qassim Region.

## Materials and methods

In the observational cross-sectional study, participants were recruited with their voluntary informed consent. The primary objectives were to comprehend the research goals and secure informed consent from participants. Researchers conducted a self-administered online survey from 20 May to 4 June 2023 among the Qassim population, using various social media platforms. Notably, Qassim, with a population of about 1.5 million, is situated in the heart of Saudi Arabia. The study included adults aged over 18 years, residing in the Qassim Region, who had voluntarily signed the informed consent before participation. Participants who did not meet these criteria were excluded.

For data collection, the researchers utilized a self-administered, predesigned, and pretested pro forma previously employed in the Kingdom of Saudi Arabia [11]. The questionnaire, designed in the Arabic language using Google Forms (Google LLC, Mountain View, California, United States), was delivered to participants through online platforms. Before answering, participants were presented with an electronic voluntary consent agreement, which they willingly accepted. To enhance the questionnaire's validity, a pilot study with 30 responses was conducted, but pilot data were not included in the final analysis. The questionnaire comprised three parts: (1) demographics, (2) general characteristics, and (3) awareness of COPD, with information obtained through closed-ended questions.

Assuming the maximum variability, which is equal to 50% (p=0.5), and taking a 95% confidence level with ±5% relative precision, the calculation for the required sample size will be as follows: Thus, using the formula n = z2pq/(pl)2 and putting in values as p=0.5 and, hence, q = 1 − 0.5 = 0.5; l = 0.05; z =1.96,

• n =(1.96)2(0.5)(0.5)/(0.5 × 0.05)2

• n = 1537

We removed 231 samples from surveys as these responses do not represent genuine preferences such as incomplete information, wrong information, etc. Thus, statistical analysis and results were done on a 1,306 sample.

Convenience sampling was utilized for the study. The gathered data were coded before being entered into a database using Microsoft Excel 2010 software (Microsoft Corporation, Redmond, Washington, United States). The data analysis was performed using SPSS Statistics version 16.0 (SPSS Inc. Released 2007. SPSS for Windows, Version 16.0. Chicago, SPSS Inc.). Descriptive statistics, such as frequency and percentage, were utilized to present the data. The statistical significance of the percentage differences was determined through significance tests like the chi-square test. In the univariate analysis, respondent awareness of COPD served as the dependent variable, while sociodemographic and behavioral factors were considered independent variables. For calculating variables, a p-value of less than 0.05 was considered statistically significant.

Ethical approval for the study was obtained from the Committee of Research Ethics, Deanship of Scientific Research, Qassim University, KSA, with the ethical approval number 23-38-12. In order to protect patient confidentiality, data was not disclosed. The research ethics committee thoroughly reviewed and approved the utilization of these confidential data in the research project. To ensure data security, all collected data was safely stored in a password-protected cloud.

## Results

A total of 1,306 participants were enrolled in the study, with only 360 (27.6%) reporting ever hearing of COPD. The majority of respondents 253 (34.5%) aged between 18 and 29 years had heard of COPD. Among the participants, singles were more prevalent (n=706) than married individuals (n=561), of whom 35.0% of singles and 18.9% of married respondents knew COPD. Approximately 56% (n=728) of those surveyed had an income of less than 5,000 SAR, and among them, nearly 31.5% (n=229) had heard of COPD. A significant proportion of respondents were students (n=541) (41.4%) and employees (n=425) (32.5%), with 38.3% and 18.8% of them, respectively, reporting ever hearing of COPD. Sociodemographic data, including age, marital status, income, and occupation, demonstrated significant associations with COPD awareness (p-value <0.05). However, gender and education level did not show significant associations with awareness of COPD (Table 1).

**Table 1:** Associations between socio-demographic variables and COPD awareness among the respondents SAR: Saudi riyal, COPD: chronic obstructive pulmonary disease

Sociodemographic variables	Category	Ever heard of COPD		Total	p-Value
		No (n=946)	Yes (n=360)		
Age	18 to 29 years	481	253	734	<0.001
		65.5%	34.5%	100.0%	
	30 to 39 years	158	42	200	
		79.0%	21.0%	100.0%	
	40 to 49 years	165	40	205	
		80.5%	19.5%	100.0%	
	50 years and above	142	25	167	
		85.0%	15.0%	100.0%	
Gender	Female	585	235	820	0.251
		71.3%	28.7%	100.0%	
	Male	361	125	486	
		74.3%	25.7%	100.0%	
Marital status	Divorced	18	6	24	<0.001
		75.0%	25.0%	100.0%	
	Married	455	106	561	
		81.1%	18.9%	100.0%	
	Single	459	247	706	
		65.0%	35.0%	100.0%	
	Widow	14	1	15	
		93.3%	6.7%	100.0%	
Monthly income in SAR	More than 15,000	112	28	140	0.002
		80.0%	20.0%	100.0%	
	10001 to 15,000	155	41	196	
		79.1%	20.9%	100.0%	
	5001 to 10,000	180	62	242	
		74.4%	25.6%	100.0%	
	Less than 5000	499	229	728	
		68.5%	31.5%	100.0%	
Education	Cannot read and write or primary school	11	3	14	0.264
		78.6%	21.4%	100.0%	
	Middle school	26	3	29	
		89.7%	10.3%	100.0%	
	Secondary school	178	74	252	
		70.6%	29.4%	100.0%	
	University	731	280	1011	
		72.3%	27.7%	100.0%	
Occupation	Employed	345	80	425	<0.001
		81.2%	18.8%	100.0%	
	Retired	89	21	110	
		80.9%	19.1%	100.0%	
	Student	334	207	541	
		61.7%	38.3%	100.0%	
	Unemployed	178	52	230	
		77.4%	22.6%	100.0%	
Do you work or study in the health field?	No	836	192	1028	<0.001
		81.3%	18.7%	100.0%	
	Yes	110	168	278	
		39.6%	60.4%	100.0%	

Out of 1,306 participants, 151 (11.6%) were smokers. The majority of smokers used cigarettes (38.4%) (n=58), and among them, 22.4% had ever heard of COPD. The second highest proportion of smokers (35.8%) (n=54) used electronic smoking devices, and 24.1% of them were aware of COPD. The lowest proportion (n=39) (25.8%) were shisha smokers, and 25.6% of them had heard of COPD. Interestingly, the majority of respondents (74%) (n=967) did not sit or spend time with smokers, yet 27.6% of them had heard of COPD. Only 230 (17.6%) of all respondents reported exposure to dust, and among those exposed, 30.9% were aware of COPD. However, smoking habits and dust exposure in relation to COPD awareness were found to be statistically insignificant with a p-value >0.05 (Table 2).

**Table 2:** Associations between smoke and dust exposure and COPD awareness COPD: chronic obstructive pulmonary disease

Smoking and other behaviors	Category	Ever heard of COPD		Total	p-value
		No	Yes		
Cigarette smoker	No	901	347	1248	0.807
		72.2%	27.8%	100.0%	
	Yes	45	13	58	
		77.6%	22.4%	100.0%	
Shisha smoker	No	917	350	1267	0.785
		72.4%	27.6%	100.0%	
	Yes	29	10	39	
		74.4%	25.6%	100.0%	
Electronic smoker	No	905	347	1252	0.558
		72.3%	27.7%	100.0%	
	Yes	41	13	54	
		75.9%	24.1%	100.0%	
Spend time or sit with a smoker	No	700	267	967	0.950
		72.4%	27.6%	100.0%	
	Yes	246	93	339	
		72.6%	27.4%	100.0%	
Spend time in shisha café	No	922	350	1272	0.807
		72.5%	27.5%	100.0%	
	Yes	24	10	34	
		70.6%	29.4%	100.0%	
Use heating devices based on charcoal burning or wood in closed places	No	812	313	1125	0.604
		72.2%	27.8%	100.0%	
	Yes	134	47	181	
		74.0%	26.0%	100.0%	
Exposed to dust, pollution, fumes, and/or chemicals at the workplace	No	787	289	1076	0.217
		73.1%	26.9%	100.0%	
	Yes	159	71	230	
		69.1%	30.9%	100.0%	
Ever diagnosed with COPD	No	941	354	1295	0.04
		72.7%	27.3%	100.0%	
	Yes	5	6	11	
		45.5%	54.5%	100.0%	

When asked about the most common symptom of COPD, the majority of respondents who had heard of COPD (58.9%, n=212/360) provided multiple options, including chest pain, fatigue, and shortness of breath. Cough, which is clinically significant, was mentioned by 12.8% of respondents. Among all respondents who had heard of COPD (n=360), 90.3% believed that cigarette smoking can lead to COPD, while 93.3% knew that quitting smoking plays a vital role in preventing COPD. Additionally, 87.5% acknowledged that smoking can exacerbate COPD, and 24.2% believed that COPD can last for more than 18 months. Furthermore, 35.5% considered COPD as a substantial financial burden for society than lung cancer, and 34.4% believed it could lead to physical disability. Only 14.4% of the 360 participants assumed that COPD is fully recoverable with short-term use of antibiotics, and 10.3% believed that COPD is a rare disease. The least number of participants (3.6%) thought that COPD is solely due to aging. All of these data related to respondents' opinions about COPD awareness appeared to be clinically significant with a p-value <0.05 (Table 3).

**Table 3:** Respondents' opinions on COPD awareness COPD: chronic obstructive pulmonary disease

Opinions	Category	Ever heard of COPD		Total	p-value
		No	Yes		
Which organ of the human body is affected by COPD?	Lung	712	292	1004	<0.001
		70.9%	29.1%	100.0%	
	Heart	61	32	93	
		65.6%	34.4%	100.0%	
	Throat	9	0	9	
		100.0%	0.0%	100.0%	
	Trachea	54	27	81	
		66.7%	33.3%	100.0%	
	I don't know	110	9	119	
		92.4%	7.6%	100.0%	
What are the most common symptoms of COPD?	Chest pain, fatigue, shortness of breath	475	212	687	<0.001
		69.1%	30.9%	100.0%	
	Shortness of breath	175	52	227	
		77.1%	22.9%	100.0%	
	Cough	99	46	145	
		68.3%	31.7%	100.0%	
	Chest pain	39	15	54	
		72.2%	27.8%	100.0%	
	I don’t know	161	32	193	
		83.4%	16.6%	100.0%	
Is COPD only due to aging?	I don't know	304	43	347	<0.001
		87.6%	12.4%	100.0%	
	No	619	304	923	
		67.1%	32.9%	100.0%	
	Yes	23	13	36	
		63.9%	36.1%	100.0%	
Is COPD more expensive for society than lung cancer?	I don't know	634	186	820	
		77.3%	22.7%	100.0%	<0.001
	No	156	46	202	
		77.2%	22.8%	100.0%	
	Yes	156	128	284	
		54.9%	45.1%	100.0%	
Does smoking cigarettes lead to COPD?	I don't know	266	26	292	<0.001
		91.1%	8.9%	100.0%	
	No	14	9	23	
		60.9%	39.1%	100.0%	
	Yes	666	325	991	
		67.2%	32.8%	100.0%	
Is COPD fully recoverable with the short-term use of antibiotics?	I don't know	589	145	734	<0.001
		80.2%	19.8%	100.0%	
	No	215	163	378	
		56.9%	43.1%	100.0%	
	Yes	142	52	194	
		73.2%	26.8%	100.0%	
Is COPD a rare disease?	I don't know	555	97	652	<0.001
		85.1%	14.9%	100.0%	
	No	196	226	422	
		46.4%	53.6%	100.0%	
	Yes	195	37	232	
		84.1%	15.9%	100.0%	
How long the COPD should last?	More than 18 months	95	87	182	<0.001
		52.2%	47.8%	100.0%	
	From 12 months to 18 months	39	35	74	
		52.7%	47.3%	100.0%	
	From 6 months to 12 months	84	44	128	
		65.6%	34.4%	100.0%	
	Less than 6 months	57	26	83	
		68.7%	31.3%	100.0%	
	I don't know	671	168	839	
		80.0%	20.0%	100.0%	
Can COPD be aggravated by smoking?	I don't know	371	41	412	<0.001
		90.0%	10.0%	100.0%	
	No	13	4	17	
		76.5%	23.5%	100.0%	
	Yes	562	315	877	
		64.1%	35.9%	100.0%	
Can COPD cause physical disability?	I don't know	592	146	738	<0.001
		80.2%	19.8%	100.0%	
	No	195	90	285	
		68.4%	31.6%	100.0%	
	Yes	159	124	283	
		56.2%	43.8%	100.0%	
Does quitting smoking play an important role in preventing COPD?	I don't know	189	20	209	<0.001
		90.4%	9.6%	100.0%	
	No	11	4	15	
		73.3%	26.7%	100.0%	
	Yes	746	336	1082	
		68.9%	31.1%	100.0%	

**Figure 1:**
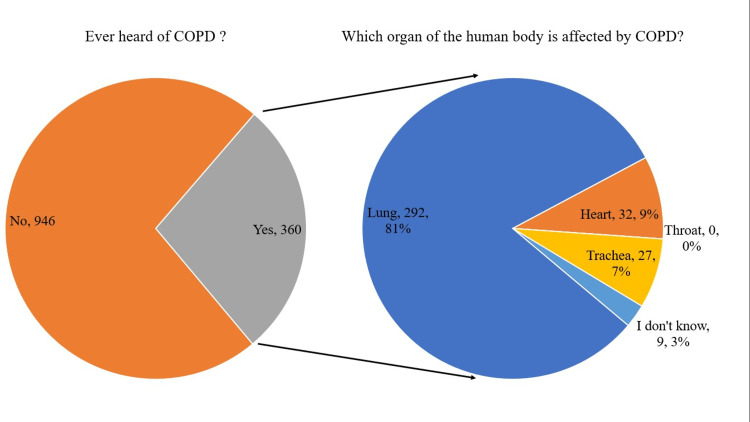
Exploring the link between COPD awareness and affected organs COPD: chronic obstructive pulmonary disease

Out of the 1,306 respondents, only 360 (27.5%) were aware of COPD. When asked about the organ affected by COPD, 292 (81%) of those who had heard of it responded correctly, stating that it affects the lungs. In contrast, 32 (8.9%) respondents selected "heart," nine (2.5%) selected "I don't know," and none selected "throat" (Figure 1).

**Figure 2:**
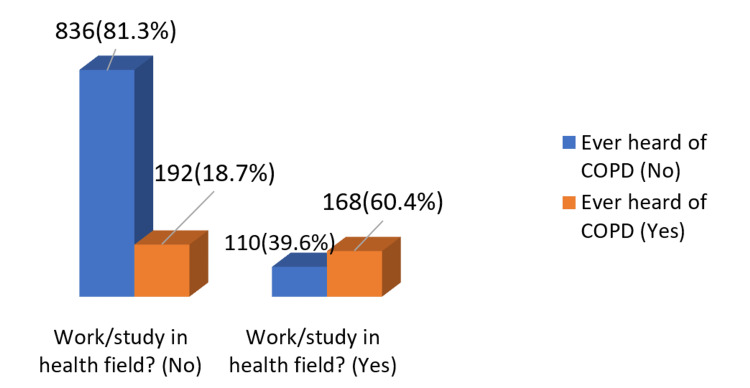
Profession or study: in the realm of health COPD: chronic obstructive pulmonary disease

Figure 2 depicts that less than one-third (27.6%) of the study population had ever heard of COPD. Notably, 168 (60.4%) of the health field workers/students were aware of COPD. In contrast, among non-health field workers/students, only 192 (18.7%) respondents had ever heard of COPD.

**Figure 3:**
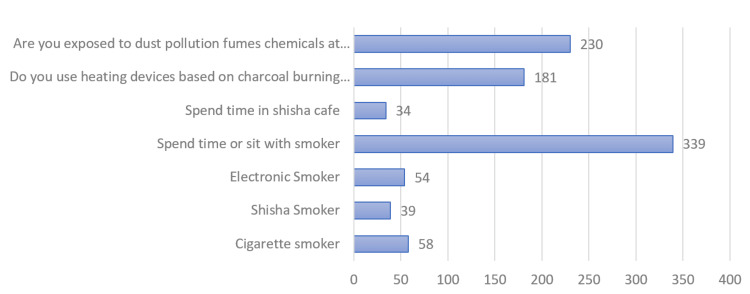
Smoke and dust exposure: distribution of study population

Out of 935 participants, 6.2% were cigarette smokers, 5.8% were electronic smokers, and 4.2% were shisha smokers. The majority of participants (36.3%) reported spending time or sitting with smokers. The second most prevalent risk factor among the participants was dust exposure (24.6%). A significant number of participants (19.4%) used heating devices based on coal, followed by those who spent time in a shisha cafe (3.6%) (Figure 3).

## Discussion

A significant deficiency in health education about COPD has been observed, not only among the general population but also among COPD patients [8] and their families [9,10]. The study's primary objective is to assess adult awareness of COPD and its risk factors. In our study, 1,306 participants were enrolled, of which 27.6% (n=360) had ever heard of COPD. Among respondents studying or working in medical-related fields, 168 (60%) were aware of COPD. However, after excluding participants from medical-related fields, the level of awareness was reduced to 18.7%.

Smoking avoidance plays a crucial role in the management and prevention of COPD, as it can slow the progressive deterioration in lung function and reduce the mortality rate among COPD patients [12]. A study involving 5,587 patients with mild COPD showed a significant improvement in lung function and better survival after 14.5 years of smoking abstinence [13]. Among our study respondents who had ever heard of COPD, 90.3% believed that cigarette smoking can lead to COPD, 93.3% knew that quitting smoking has an important role in preventing COPD, and 87.5% answered that COPD can be aggravated by smoking.

Among the Saudi population, smoking prevalence is increasing, leading to a higher prevalence of COPD [4]. A low awareness of COPD has been reported among smokers in Saudi Arabia [7], which likely contributes to the significant under-diagnosis of COPD. In our study, out of 1306 participants, 151 (11.6%) were smokers. The majority of smokers used cigarettes (38.4%). Among all cigarette smokers, 22.4% had ever heard of COPD. The second highest proportion of smokers (35.8%) used electronic smoking devices, and 25.8% were shisha smokers. However, smoking habits in relation to COPD awareness were found to be statistically insignificant in our study.

Occupational or work-related lung diseases are respiratory conditions that have been induced or exacerbated by prolonged exposure to specific irritants in the working environment. Hazardous exposures that pose a risk for occupational lung diseases encompass substances such as dust, chemicals, fungal spores, and certain animal excrement [14]. Respiratory illnesses represent the most prevalent category of occupational diseases. A study was conducted at Qassim Cement Factory to investigate the prevalence of respiratory complications, revealing that a significant number of workers with more than three years of work experience exhibited manifestations of respiratory disease. Participants' sputum samples were collected, and subsequent histopathological examination of approximately 70% of all subjects exhibited severe inflammation [15].

A meta-analysis comprising nine studies investigated the correlation between occupational dust exposure and COPD. The study provided compelling evidence supporting this association. Workers exposed to dust had a 1.51 times higher risk of developing COPD compared to controls [16]. In another study, the impact of occupational dust exposure on respiratory function in individuals with early COPD was assessed. The results from a five-year follow-up indicated a significant effect of dust exposure on the rate of lung function decline [17]. Furthermore, a separate study demonstrated that prior dust exposure was associated with a deterioration of symptoms over a one-year follow-up in patients with COPD [18]. In our study, approximately 230 respondents, accounting for around 17.6% of all participants, reported exposure to dust.

According to various geographic area-based studies, there appears to be significant variation in COPD awareness among the general population. In Slovenia, approximately 50% of the population had never heard of the term COPD [19], while in Singapore, this percentage was around 35% [20]. In Syria, the proportion of individuals unaware of COPD was 25.4% [21], and in Spain, it was 17% [21]. In France, about 8% of the participants were able to identify COPD terms [22]. In India, the awareness level of COPD was reported to be notably low at only 0.9% [23]. On the other hand, a study conducted in the Aseer Region of Saudi Arabia revealed a COPD awareness rate of 30.1% among 116 participants [11]. Interestingly, our study in the Qassim population showed a relatively similar COPD awareness level of 27.6% among 360 participants.

In the Syrian study, the awareness of COPD was reported to be 62% among 996 participants [24]. Among health field workers/students in the same study, 87.3% (n=829) had heard of COPD, while in our study, the awareness was slightly lower at 60.4%. On the other hand, for non-health field workers/students in Soriano et al.'s study (2012), the awareness rate was 25% (n=167), whereas in our study, it was 18.7% (n=192). According to Ghorpade et al.'s [23] study, the awareness of the word COPD among the Indian population was only 0.9%, whereas in our study, it was significantly higher at 27.6%. Regarding knowledge about the affected organ by COPD, Ghorpade et al.'s [23] study found that 72% of those who heard about COPD correctly identified the lungs as the affected organ, 6% thought it was the heart, and 22% did not know. In our study, a higher percentage (81%) correctly identified the lungs, but 32.9% mistakenly thought it affected the heart, and 9.3% were unsure about the affected organ.

In our study, 52.6% of all respondents assumed that "chest pain, fatigue, and shortness of breath" are the most common symptoms of COPD. Mahmood et al.'s [11] study reported that the general population's knowledge regarding the most common symptom of COPD was "cough with more symptoms" (56.03%). The negative impact of COPD symptoms on physical activity can lead to muscle deconditioning, further exacerbating dyspnea, and ultimately resulting in a deterioration of health status [25]. In our study, 21.8% of all respondents agreed that COPD can lead to physical disability.

Inhalation of smoke from tobacco products smoked by other people, as well as exposure to both secondhand and thirdhand smoke, is known as passive smoking. It is well-established that secondhand smoke can cause serious health issues, including lung cancer, heart disease, and stroke. Furthermore, it may increase the risk of inflammatory lung conditions such as COPD. In our study, 28.6% of respondents reported routinely spending time with smokers or sitting in shisha cafes, which is considered passive smoking. Passive smoking has been proven to be as equally dangerous as smoking itself and can have negative consequences on health in the future [26].

Aging is a significant risk factor for chronic lung diseases like COPD [27]. Both aging and smoking have a profound impact on the lung capability of COPD patients. Particularly, the effect of smoking appears to be modified in the presence of advanced age for older COPD patients [28]. In our study, 70.7% of all respondents did not agree that COPD is solely due to aging.

COPD does not currently have a cure, but various treatments can help control symptoms and slow the disease's progression. Treatment options include quitting smoking, using inhalers and tablets, participating in pulmonary rehabilitation programs, and in some cases, considering surgery or a lung transplant [29]. In our study, only 14.8% of all respondents assumed that COPD is fully recoverable with short-term use of antibiotics, highlighting a common misconception about the disease. Previous studies have demonstrated that COPD is associated with a significant economic burden, encompassing both direct expenses for medical services and indirect expenses for society [30]. In our study, 21.7% of all respondents thought that COPD is more expensive for society compared to lung cancer, indicating varying perceptions about the economic impact of different respiratory conditions.

It is essential to recognize and address the limitations that may impact the findings and conclusions of our study. Firstly, being a cross-sectional study, the results are representative only of a specific period of time and cannot establish causal relationships between variables. Additionally, since our research was conducted solely in the Qassim Region of Saudi Arabia, generalizing the study findings to the entire population of Saudi Arabia may not be feasible.

Furthermore, the recruitment of participants through social media may have introduced a selection bias, favoring younger individuals from higher socioeconomic classes. To mitigate this bias, we aimed to include a large sample size of 1,306 respondents, ensuring a diverse mix of subjects from various age groups and socioeconomic backgrounds. Moreover, we identified and accounted for participants with medical backgrounds by incorporating a question in the questionnaire to minimize the potential for selection bias.

To gain a comprehensive understanding of the rising prevalence of COPD among the general population, conducting a larger, cross-country study that includes multiple regions would be beneficial. Such a study could provide more robust insights into the trends and factors influencing COPD prevalence across different demographics and geographical areas.

## Conclusions

In conclusion, our study revealed a concerning lack of awareness regarding COPD among the general population in the Qassim Region of Saudi Arabia. Raising awareness about COPD is of utmost importance to facilitate early diagnosis and appropriate treatment. In this region, as well as in Saudi Arabia as a whole, COPD and its associated risk factors are highly prevalent, primarily due to exposure to air pollutants from active smoking or indoor/outdoor particle exposure. A significant proportion of respondents reported spending time around smokers, with cigarette smokers constituting the largest portion, followed by electronic smokers and shisha users. Our study highlighted that the level of COPD awareness among respondents was relatively low. Only a small percentage of participants correctly knew that COPD could lead to physical disability. To address this knowledge gap, it is essential to enhance the primary school curriculum and include COPD-related topics in public forums and awareness campaigns. It is evident that there is a pressing need for additional national research to support policymakers in developing and implementing effective preventive and therapeutic policies to combat COPD and its consequences. By increasing awareness, adopting preventive measures, and fostering a better understanding of COPD, we can work toward reducing the burden of this debilitating respiratory condition on individuals and society as a whole.
